# Basilar Artery Diameter: Establishing a Reference Range for a Selected Black African Population

**DOI:** 10.4314/ejhs.v34i5.5

**Published:** 2024-09

**Authors:** Angel-Mary C Anakwue, Afam C Umeha, Beatrice U Maduka

**Affiliations:** 1 Department of Medical Radiography and Radiological Sciences Faculty of Health Sciences and Technology, College of Medicine, University of Nigeria Enugu Campus, Enugu, Nigeria

**Keywords:** Basilar artery diameter, brain scan, computed tomography, Enugu

## Abstract

**Background:**

The basilar artery (BA) is a crucial vessel in the posterior cerebral circulation, supplying blood to the occipital lobes, cerebellum, and brainstem. Variations in BA diameter can be indicative of pathological conditions and may serve as a predictive marker for cerebrovascular events. Despite the importance of these measurements, data on the normal BA diameter in the Black African population is limited. This study aims to establish baseline values for BA diameter in healthy adults in Enugu, southeast Nigeria, and to examine any potential gender differences.

**Methods:**

A cross-sectional study was conducted on 298 subjects (150 males, 148 females) at the University of Nigeria Teaching Hospital Ituku/Ozalla, Enugu State. Participants, referred for brain CT, had normal radiologist reports. Contrast-enhanced brain CT scans were performed using a 64-slice Brilliance Philips scanner. BA diameter was measured on axial images at the midpons level.

**Results:**

The participants had a mean age of 49.4 years (±14 years). The BA diameter ranged from 3.1mm to 5.5mm, with a mean of 4.2mm (±0.5mm). Males had a significantly larger BA diameter compared to females (p<0.05). Diameter increased with age in both genders.

**Conclusions:**

This study provides reference values for BA diameter in a Nigerian population, highlighting a gender difference and age-related increase in diameter. These findings contribute to the understanding of BA geometry in Black Africans and can assist in diagnosing and managing cerebrovascular conditions.

## Introduction

The basilar artery (BA) is vital for supplying blood to critical brain regions. Its diameter is a significant marker for various cerebrovascular conditions, including atherosclerosis and aneurysms. Although global studies have reported on BA diameter, data specific to the Black African population is sparse. Understanding normal BAdimensions for this demographic is crucial, particularly given the rising incidence of stroke and cardiovascular diseases in Africa. Thisstudy seeks to fill this gap by establishing normal BA diameter values in a Nigerian population.

## Materials and Methods

This retrospective study was conducted from December 2020 to November 2022 at the Radiation Medicine Department, University of Nigeria Teaching Hospital, Enugu. Participants were Igbo adults aged 20 and above, with normal blood pressure, glucose, and lipid levels, and without BA or vertebral artery anomalies or infarcts. CT scans were performed with a Philips 64-slice Brilliance machine, and BA diameter was measured using digital calipers on axial images at the midpons level. Inter- and intra-observer reliability were confirmed with high correlation coefficients as shown in Table 1a.

**Table 1a:** Intra-observer and inter-observer reliability

Variable	Intraclass Correlation^b^	95% Confidence Interval		F Test with True Value 0			

		Lower Bound	Upper Bound	Value	df1	df2	P

		Intraclass Correlation Coefficient (within Observer)					
**Single Measures**	.965^a^	0.914	0.986	56.515	19	19	<0.001
**Average Measures**	.982^c^	0.955	0.993	56.515	19	19	<0.001
	***Intraclass Correlation Coefficient (Inter rater)***						
*Single Measures*	.918^a^	0.805	0.967	22.318	19	19	<0.001
*Average Measures*	.957^c^	0.892	0.983	22.318	19	19	<0.001

## Results

Out of 298 subjects, 150 were male and 148 were female. The average height was 1.7m and weight was 82.2kg (Table 2). The mean BA diameter was 4.2mm as shown in Table 3, with males showing larger diameters than females (4.3mm ±0.5 vs. 4.1mm ±0.4, p<0.05) as in Table 4. Diameter also increased with age (Tables 5 and 6).

**Table 2:** Subject characteristics

Variable			Gender				

			Male		Female		Total
			N (%)		N (%)		N(%)
Age group (Years)		(≤) 31	25 (8.4)		11(3.7)		36 (12.1)
		32 – 41	38(12.8)		30(10.1)		68(22.8)
		42 – 51	18(6.0)		33 (11.1)		51(17.1)
		52 – 61	33 (11.1)		34(11.4)		67(22.5)
		62+	36(12.1)		40(13.4)		76(25.5)
		Total	150(50.3)		148(49.7)		298(100.0)

				Mean	SD	Min	Max

Gender	Male	Age (Years)		48.0	14.9	21.0	78.0
		Weight (Kg)		82.8	6.8	62.0	94.5
		Height (m)		1.7	.0	1.6	1.8
		Body Mass Index (Kg/m2)		28.3	2.2	20.1	31.9
	Female	Age (Years)		50.9	13.1	22.0	75.0
		Weight (Kg)		80.6	6.0	63.0	98.5
		Height (m)		1.7	.0	1.6	1.8
		Body Mass Index (Kg/m2)		28.2	2.2	22.3	36.2
	Total	Age (Years)		49.4	14.0	21.0	78.0
		Weight (Kg)		81.7	6.5	62.0	98.5
		Height (m)		1.7	.0	1.6	1.8
		Body Mass Index (Kg/m2)		28.2	2.2	20.1	36.2

**Table 3:** Mean basilar artery diameter

Variable		Gender		

		Male	Female	Total
Basilar artery diameter (mm)	Mean	4.3	4.1	4.2
	SD	0.5	0.4	0.5
	Min	3.1	3.2	3.1
	Max	5.5	5.3	5.5
	5th Percentile	3.5	3.4	3.4
	25th Percentile	3.9	3.8	3.8
	75th Percentile	4.7	4.3	4.6
	95th Percentile	5.1	4.9	5.0

**Table 4:** Gender difference in the diameter of the BA

variable			Equal variances	

			Assumed	Not assumed
Levene's Test for Equality of Variances	F		8.234	
	Sig.		0.004	
t-test for Equality of Means	T		4.017	4.021
	Df		296	289.987
	Sig. (2-tailed)		0.000	0.000
	Mean Difference		0.2242	0.2242
	Std. Error Difference		0.0558	0.0557
	95% CI of	Lower	0.1143	0.1144
	Difference	Upper	0.3340	0.3339

**Table 5:** Basilar artery diameter for the different age ranges

Variable		Basilar artery diameter (mm)			

		Mean	SD	Min	Max
Age group (Years)	<= 31	3.8	0.4	3.1	4.4
	32 - 41	4.0	0.4	3.3	5.0
	42 - 51	4.2	0.4	3.3	5.0
	52 - 61	4.3	0.4	3.4	5.1
	62+	4.5	0.5	3.4	5.5

**Table 6:** Age difference in basilar artery diameter

Gender				Equal variances	

				Assumed	Not assumed
Male	Levene's Test for Equality of Variances t-test for Equality of Means	F		0.076	
		Sig.		0.783	
		T		7.292	7.292
		Df		148	147.034
		Sig. (2-tailed)		0.000	0.000
		Mean Difference		0.5312	0.5312
		Std. Error Difference		0.0728	0.0728
		95% Confidence Interval of	Lower	0.3873	0.3872
		the Difference	Upper	0.6751	0.6752
Female	Levene's Test for Equality of Variances t-test for Equality of Means	F		0.845	
		Sig.		0.359	
		T		4.755	4.793
		Df		146	145.954
		Sig. (2-tailed)		0.000	0.000
		Mean Difference		0.3233	0.3233
		Std. Error Difference		0.0680	0.0675
		95% Confidence Interval of the Difference	Lower	0.1889	0.1900
			Upper	0.4577	0.4566

Table 1 shows that the measurements obtained were reliable and reproducible within and between the observers.

Table 2 showed that the participants aged 62 years ≥ constituted the highest proportion of participants (n=76; 25.5%) followed by subjects aged between 32 and 41 years (n=68; 22.8%). The mean age of the male participants was 48 years while that of the females was 50.9 years respectively. The total mean BMI for males was 28.3kg/m2 and that of females was 28.2kg/m2.

The mean basilar artery diameter is 4.3±0.5 mm for males and 4.1±0.4mm for females. The percentile range for 5^th^ and 95^th^ percentile is 3.5mm and 5.1mm for males and 3.4mm and 4.9 mm for females. This constituted the normal range for the population studied.

An independent samples t-test was run to determine if there was a significant difference between the BAD measurements of males and females. This is shown in the table above. Levene's test disclosed that equal variances for each gender was not assumed (F = 8.234, p = 0.004). With a t-value of 4.021 and p value of less than 0.05, there was a significant difference between the BAD measurements of males and females.

The BAD increased across all groups, from an average value of 3.8mm to 4.5mm.

Results show that, controlling for gender, there is a significant difference between the BAD measurements for subjects below and above 50 years for both gender (males: t = 7.292, p<0.05; females: t = 4.755, p<0.05).

## Discussion

This study's findings align with previous research indicating variations in BA diameter by race and gender. The observed diameter range (3.1mm to 5.5mm) differs from other studies, which may be due to genetic or regional factors. Establishing local reference values is critical for accurate diagnosis and treatment of cerebrovascular diseases in the Nigerian population. Future studies should consider including additional imaging techniques and exploring other ethnic groups in Nigeria.

In conclusion, this research establishes normal BA diameter values for a Nigerian population, revealing gender differences and age-related variations. These reference values are essential for accurate diagnosis and management of cerebrovascular conditions and may help prevent misinterpretations based on values derived from other populations.
